# 1,3-Diphenylisobenzofuran

**DOI:** 10.1107/S1600536808006016

**Published:** 2008-03-12

**Authors:** René T. Boeré, Peter W. Dibble, Kristapher E. Fischer

**Affiliations:** a Department of Chemistry and Biochemistry, University of Lethbridge, Lethbridge, AB, Canada T1K 3M4

## Abstract

The structure of the title compound, 1,3-diphenyl-2-benzofuran, C_20_H_14_O, exhibits a distinct alternation of short [mean 1.361 (3) Å] and long [mean 1.431 (3) Å] C—C bonds around the benzofuran ring system, indicating a predominantly polyene character. Over 60 Diels–Alder adducts of this commercially available furan have been structurally characterized, but this is the first report of the structure of the parent compound.

## Related literature

For related literature, see: Wege (1998 ▶); Friedrichsen (1980 ▶); Friedrichsen (1999 ▶); Allen (2002 ▶); Yang & Duan (1991 ▶); Rodrigo *et al.* (1986 ▶); Lynch *et al.* (1995 ▶): Lu *et al.* (2006 ▶).
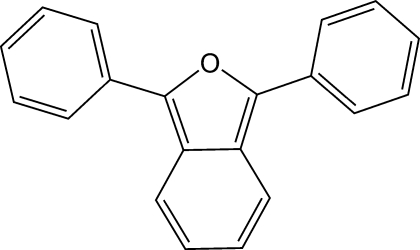


## Experimental

### 

#### Crystal data

C_20_H_14_O
*M* *_r_* = 270.31
Monoclinic, 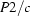

*a* = 12.8198 (17) Å
*b* = 5.5273 (8) Å
*c* = 19.945 (3) Å
β = 106.480 (2)°
*V* = 1355.2 (3) Å^3^
*Z* = 4
Mo *K*α radiation
μ = 0.08 mm^−1^
*T* = 173 (2) K
0.28 × 0.13 × 0.04 mm

#### Data collection

Bruker APEXII CCD area-detector diffractometer
Absorption correction: multi-scan (*SADABS*; Sheldrick, 2004 ▶) *T*_min_ = 0.977, *T*_max_ = 0.998
13976 measured reflections
2854 independent reflections
1664 reflections with *I* > 2σ(*I*)
*R*_int_ = 0.085

#### Refinement

*R*[*F* ^2^ > 2σ(*F* ^2^)] = 0.047
*wR*(*F* ^2^) = 0.105
*S* = 1.03
2854 reflections
191 parameters
H-atom parameters constrained
Δρ_max_ = 0.20 e Å^−3^
Δρ_min_ = −0.21 e Å^−3^

### 

Data collection: *APEX2* (Bruker, 2006 ▶); cell refinement: *APEX2*; data reduction: *SAINT* (Bruker, 2006 ▶); program(s) used to solve structure: *SHELXTL* (Sheldrick, 2008 ▶); program(s) used to refine structure: *SHELXTL*; molecular graphics: *SHELXTL*; software used to prepare material for publication: *publCIF* (Westrip, 2008 ▶).

## Supplementary Material

Crystal structure: contains datablocks global, I. DOI: 10.1107/S1600536808006016/pv2071sup1.cif

Structure factors: contains datablocks I. DOI: 10.1107/S1600536808006016/pv2071Isup2.hkl

Additional supplementary materials:  crystallographic information; 3D view; checkCIF report

## Figures and Tables

**Table 1:** Selected bond lengths (Å)

O1—C1	1.366 (2)
O1—C8	1.369 (2)
C1—C2	1.373 (3)
C2—C3	1.425 (3)
C2—C7	1.435 (3)
C3—C4	1.351 (3)
C4—C5	1.435 (3)
C5—C6	1.346 (3)
C6—C7	1.427 (3)
C7—C8	1.372 (3)

**Fig. 1.**
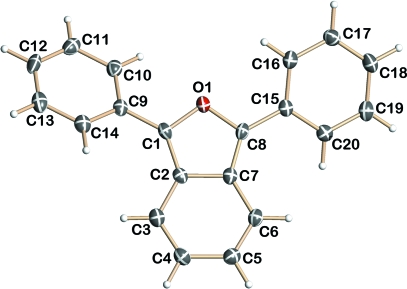
Thermal ellipsoid plot of the structure of (I) drawn at 50% probability level showing the atom numbering scheme.

## supplementary crystallographic information

### Comment

Isobenzofuran is a ten π-electron system exhibiting very high reactivity in Diels-Alder reactions (Wege, 1998; Friedrichsen, 1999). The commercially available 1,3-diphenyl-2-benzofuran, (I), is a molecule with many interesting features (Friedrichsen, 1980) and unlike the parent compound is stable in the solid state. It is brightly fluorescent, electroluminescent and, despite its stability relative to isobenzofuran is still highly reactive in Diels-Alder reactions. Its reactivity is exploited in the quantitative kinetic investigations of biological singlet oxygen generation and in the *in situ* trapping of transient olefin intermediates. With respect to this latter application, the high reactivity of (I) and the crystallinity of its adducts account for the sixty-eight X-ray structures of diphenylisobenzofuran adducts that appear in the Cambridge Crystallographic Database (Allen, 2002). Surprisingly, an X-ray structure of (I) has not appeared in the literature.

Calculations have suggested that isobenzofuran has a low resonance energy (Yang & Duan, 1991) and mainly polyene character. The structures of isobenzofurans are of interest since the degree of bond length alternation serves to indicate the balance between aromatic and polyene character. Only three structures of isobenzofurans have been published previously: 1-cyano-4,5-methylenedioxyisobenzofuran (Rodrigo *et al.*, 1986); 3,6-dimethoxyisobenzofuran (Lynch *et al.*, 1995) and the highly strained 9,10,12,13-tetraphenyl-11-oxacyclopenta[*b*]triphenylene (Lu *et al.*, 2006)*.* All three structures show that the isobenzofuran core is essentially polyene in character with the structure of the furan ring very similar to that of furan itself.

The structure of (I) (Fig. 1) is very closely comparable to those of the three previously published examples. There is no significant evidence of bond length averaging indicating that it has predominantly polyene character. Of the three published structures, the bond lengths of (I) are closest to those calculated for the parent compound using the MP2/6–31G* basis set (Friedrichsen, 1980). The only noticeable difference is in the C1—C2, C7—C87 bonds which are slightly longer, perhaps the effect of conjugation to the phenyl substitutents. While the most recent structure (Lu *et al.*, 2006) contains the diphenylisobenzofuran substructure, extensive *peri* interactions throughout the molecule lead to significant deviations from planarity making direct structural comparisons less meaningfull.

Strong steric interactions between the phenyl substituents of (I) and the *peri* H atoms (H3 and H6) are indicated by a 25° torsional twist of the phenyl rings out of the plane of the isobenzofuran ring and wide 135.3 (2)° and 135.5 (2)° bond angles for C2—C1—C9, C7—C8—C15.

### Experimental

Commercial 1,3-diphenyl-2-benzofuran [CAS-5471–66–6] (Aldrich) was recrystallized from ethanol.

### Refinement

The space group was determined by trial and error and confirmed by a sucessful refinement. The alternative choice, *P*c, gave unrealistic geometrical parameters. H-atoms were included at geometrically idealized positions with C—H distance 0.95 Å and *U*_iso_ = 1.2 times *U*_eq_ of the C-atoms to which they were bonded. The model was refined to convergence, and there were no chemically significant features on the final difference Fourier map.

### Crystal data

**Table:**

C_20_H_14_O	*F*_000_ = 568
*M**_r_* = 270.31	*D*_x_ = 1.325 Mg m^−^^3^
Monoclinic, *P*2/*c*	Melting point: 128 K
Hall symbol: -P 2yc	Mo *K*α radiation λ = 0.71073 Å
*a* = 12.8198 (17) Å	Cell parameters from 2945 reflections
*b* = 5.5273 (8) Å	θ = 2.3–24.1º
*c* = 19.945 (3) Å	µ = 0.08 mm^−^^1^
β = 106.480 (2)º	*T* = 173 (2) K
*V* = 1355.2 (3) Å^3^	Plate, green
*Z* = 4	0.28 × 0.13 × 0.04 mm

### Data collection

**Table:**

Bruker APEXII CCD area-detector diffractometer	2854 independent reflections
Monochromator: graphite	1664 reflections with *I* > 2σ(*I*)
*T* = 173(2) K	*R*_int_ = 0.085
*P* = 95 kPa	θ_max_ = 26.7º
φ and ω scans	θ_min_ = 2.1º
Absorption correction: multi-scan(SADABS; Sheldrick, 2004)	*h* = −16→16
*T*_min_ = 0.977, *T*_max_ = 0.998	*k* = −6→6
13976 measured reflections	*l* = −25→25

### Refinement

**Table:**

Refinement on *F*^2^	Hydrogen site location: inferred from neighbouring sites
Least-squares matrix: full	H-atom parameters constrained
*R*[*F*^2^ > 2σ(*F*^2^)] = 0.047	*w* = 1/[σ^2^(*F*_o_^2^) + (0.0358*P*)^2^ + 0.3395*P*] where *P* = (*F*_o_^2^ + 2*F*_c_^2^)/3
*wR*(*F*^2^) = 0.105	(Δ/σ)_max_ < 0.001
*S* = 1.03	Δρ_max_ = 0.20 e Å^−^^3^
2854 reflections	Δρ_min_ = −0.21 e Å^−^^3^
191 parameters	Extinction correction: SHELXTL (Sheldrick, 2008), Fc^*^=kFc[1+0.001xFc^2^λ^3^/sin(2θ)]^-1/4^
Primary atom site location: structure-invariant direct methods	Extinction coefficient: 0.0103 (12)
Secondary atom site location: difference Fourier map	

### Special details

**Table:**

Geometry. All e.s.d.'s (except the e.s.d. in the dihedral angle between two l.s. planes) are estimated using the full covariance matrix. The cell e.s.d.'s are taken into account individually in the estimation of e.s.d.'s in distances, angles and torsion angles; correlations between e.s.d.'s in cell parameters are only used when they are defined by crystal symmetry. An approximate (isotropic) treatment of cell e.s.d.'s is used for estimating e.s.d.'s involving l.s. planes.
Refinement. Refinement of *F*^2^ against ALL reflections. The weighted *R*-factor *wR* and goodness of fit *S* are based on *F*^2^, conventional *R*-factors *R* are based on *F*, with *F* set to zero for negative *F*^2^. The threshold expression of *F*^2^ > σ(*F*^2^) is used only for calculating *R*-factors(gt) *etc*. and is not relevant to the choice of reflections for refinement. *R*-factors based on *F*^2^ are statistically about twice as large as those based on *F*, and *R*- factors based on ALL data will be even larger. All e.s.d.'s (except the e.s.d. in the dihedral angle between two l.s. planes) are estimated using the full covariance matrix. The cell e.s.d.'s are taken into account individually in the estimation of e.s.d.'s in distances, angles and torsion angles; correlations between e.s.d.'s in cell parameters are only used when they are defined by crystal symmetry. An approximate (isotropic) treatment of cell e.s.d.'s is used for estimating e.s.d.'s involving l.s. planes.

### Fractional atomic coordinates and isotropic or equivalent isotropic displacement parameters (Å^2^)

**Table:**

	*x*	*y*	*z*	*U*_iso_*/*U*_eq_	
O1	0.29644 (11)	0.9505 (2)	0.35614 (7)	0.0205 (4)	
C1	0.21954 (16)	0.8022 (4)	0.31456 (11)	0.0202 (5)	
C2	0.20930 (16)	0.8560 (4)	0.24577 (11)	0.0196 (5)	
C3	0.14208 (17)	0.7650 (4)	0.18104 (11)	0.0218 (5)	
H3	0.0905	0.6406	0.1801	0.026*	
C4	0.15333 (17)	0.8597 (4)	0.12105 (12)	0.0240 (5)	
H4	0.1089	0.8002	0.0776	0.029*	
C5	0.23051 (17)	1.0476 (4)	0.12106 (11)	0.0245 (5)	
H5	0.2363	1.1087	0.0777	0.029*	
C6	0.29489 (17)	1.1392 (4)	0.18114 (11)	0.0234 (5)	
H6	0.3461	1.2628	0.1804	0.028*	
C7	0.28490 (16)	1.0474 (4)	0.24591 (11)	0.0191 (5)	
C8	0.33653 (16)	1.0998 (4)	0.31456 (11)	0.0203 (5)	
C9	0.16786 (16)	0.6343 (4)	0.35090 (11)	0.0187 (5)	
C10	0.16433 (16)	0.6805 (4)	0.41877 (11)	0.0236 (5)	
H10	0.1966	0.8240	0.4418	0.028*	
C11	0.11450 (17)	0.5203 (4)	0.45328 (12)	0.0256 (5)	
H11	0.1140	0.5527	0.5000	0.031*	
C12	0.06547 (17)	0.3130 (4)	0.41987 (11)	0.0265 (6)	
H12	0.0300	0.2046	0.4432	0.032*	
C13	0.06827 (17)	0.2643 (4)	0.35262 (12)	0.0246 (5)	
H13	0.0349	0.1216	0.3297	0.030*	
C14	0.11927 (16)	0.4218 (4)	0.31841 (11)	0.0214 (5)	
H14	0.1214	0.3856	0.2723	0.026*	
C15	0.42170 (17)	1.2651 (4)	0.35097 (11)	0.0204 (5)	
C16	0.48555 (17)	1.2125 (4)	0.41833 (11)	0.0241 (5)	
H16	0.4722	1.0692	0.4409	0.029*	
C17	0.56847 (17)	1.3671 (4)	0.45278 (12)	0.0273 (6)	
H17	0.6113	1.3302	0.4989	0.033*	
C18	0.58898 (18)	1.5752 (4)	0.42018 (12)	0.0285 (6)	
H18	0.6464	1.6803	0.4436	0.034*	
C19	0.52636 (17)	1.6294 (4)	0.35396 (12)	0.0281 (6)	
H19	0.5407	1.7721	0.3316	0.034*	
C20	0.44235 (17)	1.4777 (4)	0.31940 (12)	0.0243 (5)	
H20	0.3985	1.5189	0.2739	0.029*	

### Atomic displacement parameters (Å^2^)

**Table:**

	*U*^11^	*U*^22^	*U*^33^	*U*^12^	*U*^13^	*U*^23^
O1	0.0184 (8)	0.0201 (8)	0.0231 (8)	−0.0025 (6)	0.0062 (7)	0.0010 (7)
C1	0.0161 (11)	0.0185 (12)	0.0258 (13)	−0.0009 (9)	0.0058 (10)	−0.0030 (10)
C2	0.0163 (11)	0.0172 (11)	0.0257 (13)	0.0014 (9)	0.0065 (10)	−0.0009 (9)
C3	0.0190 (12)	0.0185 (11)	0.0272 (13)	0.0002 (9)	0.0055 (10)	−0.0018 (10)
C4	0.0213 (12)	0.0256 (13)	0.0248 (13)	0.0016 (10)	0.0059 (10)	−0.0023 (10)
C5	0.0231 (12)	0.0249 (12)	0.0266 (13)	0.0033 (10)	0.0090 (10)	0.0046 (10)
C6	0.0207 (12)	0.0219 (12)	0.0281 (13)	0.0003 (10)	0.0076 (10)	0.0023 (10)
C7	0.0185 (11)	0.0179 (11)	0.0228 (12)	0.0027 (9)	0.0089 (10)	0.0002 (9)
C8	0.0209 (12)	0.0173 (12)	0.0252 (13)	0.0020 (9)	0.0105 (10)	0.0040 (9)
C9	0.0145 (10)	0.0178 (11)	0.0233 (12)	0.0027 (9)	0.0046 (9)	0.0032 (9)
C10	0.0203 (12)	0.0226 (12)	0.0265 (13)	−0.0024 (9)	0.0045 (10)	−0.0001 (10)
C11	0.0258 (12)	0.0276 (13)	0.0235 (12)	−0.0004 (10)	0.0071 (10)	0.0019 (10)
C12	0.0254 (13)	0.0249 (13)	0.0308 (14)	−0.0011 (10)	0.0107 (11)	0.0064 (11)
C13	0.0196 (12)	0.0196 (12)	0.0342 (14)	0.0005 (10)	0.0067 (11)	0.0020 (10)
C14	0.0183 (11)	0.0211 (12)	0.0239 (12)	0.0038 (9)	0.0045 (10)	0.0019 (10)
C15	0.0168 (11)	0.0196 (12)	0.0265 (13)	0.0014 (9)	0.0092 (10)	−0.0031 (10)
C16	0.0228 (12)	0.0232 (13)	0.0271 (13)	−0.0021 (10)	0.0084 (10)	−0.0038 (10)
C17	0.0203 (12)	0.0334 (14)	0.0277 (13)	0.0002 (10)	0.0060 (10)	−0.0088 (11)
C18	0.0216 (12)	0.0260 (14)	0.0397 (15)	−0.0069 (10)	0.0119 (11)	−0.0146 (11)
C19	0.0275 (13)	0.0188 (13)	0.0428 (16)	−0.0005 (10)	0.0178 (12)	−0.0031 (11)
C20	0.0228 (12)	0.0211 (12)	0.0313 (13)	0.0025 (10)	0.0113 (10)	−0.0001 (10)

### Geometric parameters (Å, °)

**Table:**

O1—C1	1.366 (2)	C10—H10	0.9500
O1—C8	1.369 (2)	C11—C12	1.384 (3)
C1—C2	1.373 (3)	C11—H11	0.9500
C1—C9	1.449 (3)	C12—C13	1.379 (3)
C2—C3	1.425 (3)	C12—H12	0.9500
C2—C7	1.435 (3)	C13—C14	1.380 (3)
C3—C4	1.351 (3)	C13—H13	0.9500
C3—H3	0.9500	C14—H14	0.9500
C4—C5	1.435 (3)	C15—C16	1.391 (3)
C4—H4	0.9500	C15—C20	1.394 (3)
C5—C6	1.346 (3)	C16—C17	1.385 (3)
C5—H5	0.9500	C16—H16	0.9500
C6—C7	1.427 (3)	C17—C18	1.383 (3)
C6—H6	0.9500	C17—H17	0.9500
C7—C8	1.372 (3)	C18—C19	1.370 (3)
C8—C15	1.451 (3)	C18—H18	0.9500
C9—C10	1.391 (3)	C19—C20	1.384 (3)
C9—C14	1.399 (3)	C19—H19	0.9500
C10—C11	1.384 (3)	C20—H20	0.9500
			
C1—O1—C8	108.91 (16)	C12—C11—C10	120.1 (2)
O1—C1—C2	108.95 (18)	C12—C11—H11	120.0
O1—C1—C9	115.74 (18)	C10—C11—H11	120.0
C2—C1—C9	135.3 (2)	C13—C12—C11	119.8 (2)
C1—C2—C3	133.7 (2)	C13—C12—H12	120.1
C1—C2—C7	106.53 (18)	C11—C12—H12	120.1
C3—C2—C7	119.77 (19)	C12—C13—C14	120.3 (2)
C4—C3—C2	118.5 (2)	C12—C13—H13	119.8
C4—C3—H3	120.8	C14—C13—H13	119.8
C2—C3—H3	120.8	C13—C14—C9	120.8 (2)
C3—C4—C5	121.8 (2)	C13—C14—H14	119.6
C3—C4—H4	119.1	C9—C14—H14	119.6
C5—C4—H4	119.1	C16—C15—C20	118.5 (2)
C6—C5—C4	121.4 (2)	C16—C15—C8	120.30 (19)
C6—C5—H5	119.3	C20—C15—C8	121.2 (2)
C4—C5—H5	119.3	C17—C16—C15	120.6 (2)
C5—C6—C7	118.8 (2)	C17—C16—H16	119.7
C5—C6—H6	120.6	C15—C16—H16	119.7
C7—C6—H6	120.6	C18—C17—C16	120.1 (2)
C8—C7—C6	133.4 (2)	C18—C17—H17	119.9
C8—C7—C2	106.96 (18)	C16—C17—H17	119.9
C6—C7—C2	119.64 (19)	C19—C18—C17	119.8 (2)
O1—C8—C7	108.65 (18)	C19—C18—H18	120.1
O1—C8—C15	115.80 (18)	C17—C18—H18	120.1
C7—C8—C15	135.5 (2)	C18—C19—C20	120.5 (2)
C10—C9—C14	118.14 (19)	C18—C19—H19	119.7
C10—C9—C1	121.01 (19)	C20—C19—H19	119.7
C14—C9—C1	120.86 (19)	C19—C20—C15	120.4 (2)
C11—C10—C9	120.9 (2)	C19—C20—H20	119.8
C11—C10—H10	119.5	C15—C20—H20	119.8
C9—C10—H10	119.5		
			
C8—O1—C1—C2	−0.2 (2)	C2—C1—C9—C10	−155.0 (2)
C8—O1—C1—C9	−179.21 (16)	O1—C1—C9—C14	−156.91 (17)
O1—C1—C2—C3	−178.8 (2)	C2—C1—C9—C14	24.5 (4)
C9—C1—C2—C3	−0.2 (4)	C14—C9—C10—C11	0.2 (3)
O1—C1—C2—C7	0.2 (2)	C1—C9—C10—C11	179.72 (19)
C9—C1—C2—C7	178.9 (2)	C9—C10—C11—C12	−1.2 (3)
C1—C2—C3—C4	−179.7 (2)	C10—C11—C12—C13	1.3 (3)
C7—C2—C3—C4	1.4 (3)	C11—C12—C13—C14	−0.3 (3)
C2—C3—C4—C5	0.0 (3)	C12—C13—C14—C9	−0.7 (3)
C3—C4—C5—C6	−0.4 (3)	C10—C9—C14—C13	0.7 (3)
C4—C5—C6—C7	−0.5 (3)	C1—C9—C14—C13	−178.76 (19)
C5—C6—C7—C8	−179.9 (2)	O1—C8—C15—C16	23.8 (3)
C5—C6—C7—C2	1.8 (3)	C7—C8—C15—C16	−154.2 (2)
C1—C2—C7—C8	−0.2 (2)	O1—C8—C15—C20	−156.92 (18)
C3—C2—C7—C8	179.07 (19)	C7—C8—C15—C20	25.1 (4)
C1—C2—C7—C6	178.52 (19)	C20—C15—C16—C17	−0.7 (3)
C3—C2—C7—C6	−2.3 (3)	C8—C15—C16—C17	178.64 (19)
C1—O1—C8—C7	0.1 (2)	C15—C16—C17—C18	−0.4 (3)
C1—O1—C8—C15	−178.40 (16)	C16—C17—C18—C19	0.7 (3)
C6—C7—C8—O1	−178.4 (2)	C17—C18—C19—C20	0.2 (3)
C2—C7—C8—O1	0.0 (2)	C18—C19—C20—C15	−1.3 (3)
C6—C7—C8—C15	−0.3 (4)	C16—C15—C20—C19	1.5 (3)
C2—C7—C8—C15	178.1 (2)	C8—C15—C20—C19	−177.77 (19)
O1—C1—C9—C10	23.6 (3)		
